# Thermally Stable HfO_2_-Based Ferroelectric Transistors for CMOS-Compatible Energy-Efficient Neuromorphic Integrated Circuits

**DOI:** 10.3390/nano16150927

**Published:** 2026-07-28

**Authors:** Fedor V. Tikhonenko, Mikhail Tarkov, Vladimir P. Popov, Andrey V. Miakonkikh, Konstantin V. Rudenko

**Affiliations:** 1 Rzhanov Institute of Semiconductor Physics SB RAS, 13, Lavrentiev Avenue, 630090 Novosibirsk, Russia; ftikhonenko@gmail.com (F.V.T.); tarkov@isp.nsc.ru (M.T.); 2 National Research Center “Kurchatov Institute”—Valiev Division of Physical and Technological Research, 36/1, Nakhimovsky Avenue, 117218 Moscow, Russia; miakonkikh@ftian.ru (A.V.M.); rudenko@ftian.ru (K.V.R.)

**Keywords:** silicon–ferroelectric–silicon structures, HAO and HZAO nanolaminates, residual polarization, coercive field, analog content addressable memory

## Abstract

HfO_2_ based thin-film ferroelectrics are metastable at room temperature and transited to the dielectric monoclinic phase upon heating. The thermal stability of such ferroelectrics increases when thin-film oxides are buried (BOX) in silicon–ferroelectric–silicon (SFS) structures formed by SmartCut^®^, where thin ferroelectric layers are stabilized by oxygen vacancies and tensile stresses in the BOX, which is similar to silicon-on-insulator (SOI) structures. The main characteristics of the ferroelectrics in MFS and SFS structures are residual polarization P_r_ and coercive field E_c_, which are determined by the fraction of the metastable ferroelectric phases that are also stabilized due to the inserted Al impurity in HfO_2_:Al_2_O_3_ 10:1 (HAO) and (HfO_2_:ZrO_2_):Al_2_O_3_ (1:1)_5_:1 (HZAO) nanolaminates. SFS structures and SFS CMOS ICs were tested after all thermal treatments at temperatures 900–1000 °C with t_BOX_ = 10–20 nm (or equivalent oxide thickness EOT = 1–2 nm) in an industrial process as gate insulators for CMOS and dual-gate DG SFS transistors. Their characteristics simulated in TCAD Sentaurus and analytic models in LTspice are investigated for an analog content addressable memory (ACAM).

## 1. Introduction

In recent years, the shortage of dynamic random-access memory (DRAM) has become a problem in the development of the global economy. This shortage is due to the rapid growth in the use of DRAM in big data and artificial intelligence (AI) processing systems [1,2,3]. A more global problem is the explosive growth in the energy consumption of data centers to 945 Twatt·hour, which is caused by the needs of AI and machine learning [4]. Energy costs alone exceed USD 100 million for a ChatGPT-4 training course with 25,000 Nvidia H100 GPUs with the DRAM consuming 17 Megawatts [3]. More than 13 TWatt·hours of electricity per year is required for GPUs to train and continuously transfer information between the processor and memory for the von Neumann computing architecture with the increasing complexity of AI models as well as the number and size of data centers. In total, sending information takes up to 95% of energy consumption. Reducing the energy consumption of integrated circuits (ICs) by orders of magnitude by abandoning the von Neumann architecture and moving to energy-efficient deep neural network ICs (DNN ICs), simulating the brain work with information processing directly in the memory, seems today to be the only way to solve the energy consumption problems of the big data economy [5,6].

The main difference between a non-volatile memory (NVM) on ferroelectrics and all other NVM types of information storage (magnetic, optical, phase) is associated with the extremely low energy consumption per operation ~ 1 fJ/op, which makes it comparable in efficiency to the human brain [6,7]. The discovery a decade and a half ago of the ferroelectric properties of ultrathin HfO_2_ films compatible with the CMOS technology doped (Al, Si, Zr, Y, La, etc.) or containing oxygen vacancies V_O_ opens the way to the mass production of an FE memory compatible with the CMOS technology under modern IC technology nodes [8,9,10].

The disadvantage of ferroelectric (FE) films based on HfO_2_ is the relatively low temperature stability of ferroelectric properties (usually up to 600 °C) as well as the formation of interlayer dielectrics (IL) at heterointerfaces with metals and silicon during a post-process heat treatment due to their oxidation by oxygen atoms from the HfO_2_ layer [11,12,13,14].

An increase in the concentration of oxygen vacancies V_O_ at interfaces stabilizes the ferroelectric phases of HfO_2_, reduces the coercive field E_c,_, increases the polarization charge density Q_r_ and, accordingly, increases the residual polarization field P_r_(V), FE hysteresis and the memory window MW of the device for external voltage V [15,16,17,18,19]. But, after removing the external voltage, a depolarization field P_d_ opposite to the Pr field appears due to Debye or Thomas–Fermi screening and IL dielectrics at heterointerfaces with a semiconductor or metal [20,21,22]. The depolarization field P_d_ leads to a decrease in residual polarization with the increasing storage time due to the repolarization of individual domains in the FE layer and, ultimately, to the loss of information in the device. The domain structure of the FE HfO_2_ layer itself increases the spread of E_c_ when the FE region sizes are close to the domain sizes [20].

Doping hafnium dioxide with metal impurities (Zr, V, Y, La, etc.) provides the highest polarization charge values Pr = 40–60 µC/cm^2^, but it leads to accelerated depolarization, a decrease in storage (retention) time <10 years, and a decrease in the number of write–read cycles (endurance parameter) to 10^9^–10^11^ for the FE device [23,24,25,26,27,28]. When doped with Al and Si impurities, the polarization P = 10–20 µC/cm^2^ and the memory window MW is noticeably smaller, but the retention time and reliability of information storage are longer [8,23,29,30,31,32,33,34,35].

The drawbacks of low thermal stability; spread of E_c_ and, therefore, thresholds of ferroelectric transistors (FeFETs); and the reduction in memory window parameters, storage time and endurance can be solved by changing the design of ferroelectric devices and moving from MFM, MFIS and MIFIS structures due to the oxidation of metals to SFS stacks with semiconductors instead of metals that are less susceptible to oxidation and the generation of excess vacancies in the FE layer. The advantages of SFS structures due to the double gate (DG) of FeFET transistors in solving the problems listed above are discussed in [34,35,36,37,38].

Using DG FeFETs on SFS substrates to dynamically control transistor thresholds instead of back gate bias voltage can avoid the use of multiple power supplies and increase the energy efficiency of low-voltage ICs [39,40]. Moreover, the ability to independently adjust the DG FeFET thresholds in ICs opens up a new functionality of SFS structures previously unattainable for the NVM memory—namely, the capability of self-healing, dragonfly prey interception and homeostasis [41]. This is based on an efficient hardware implementation of synapse with astrocytes/dendrites, which will complement the existing neuromorphic equipment.

The purpose of this paper was to optimize the design of the FE device for NN IC cross-bar matrices as well as the structure and composition of FE nanolayers to increase the thermal stability of ferroelectric hysteresis after all thermal treatments up to maximum temperatures of 900 °C for HZAO to 950 °C for HAO and to make it compatible with the modern silicon CMOS process flow. The results of previous studies indicate the thermal stability of multilayer nanometer HfO_2_ and ZrO_2_ insulators laminated in ALD-supercycles with several aluminum oxide Al_2_O_3_ monolayers [42,43]. The convenience of using these materials for CMOS technology for logic FET and FeFET integration in the cross-bar matrix cells is obvious. The fabrication, measurements, and analytical and model calculations (TCAD, BSIM-IMG, LTSPICE) of the properties of ferroelectric capacitors (FECAPs), tunnel diodes (FTJ) and transistors (FE FET) will provide the choice of thermally stable FE devices for cross-bar NN IC matrices. Proving the thermal stability of these multilayer nanometer-scale HfO_2_ and ZrO_2_ insulators with alumina in SFS structures in an industrial CMOS process is a crucial step for the future fabrication of energy-efficient neural network-based integrated circuits.

## 2. Materials and Methods of SFS Heterostructure Formation

To manufacture SFS structures, *n*/*p*-type wafers with the (001) orientation and a resistivity of 5–10 Ohm cm were used. A pair of Si wafers were chemically treated in SC1 and SC2 solutions before bonding. Then, after removing the natural oxide in diluted hydrofluoric acid, the wafer surfaces were treated sequentially in oxygen and nitrogen plasma at substrate temperatures up to 500 °C to form interlayer silicon oxynitride SiON as a protective dielectric with a thickness about 2.0 nm and prevent a further oxidation of silicon wafers during post-process treatments. Ferroelectric nanolaminated layers with a total thickness of 10–20 nm of HfO_2_:Al_2_O_3_ 10:1 (HAO) or (HfO_2_:ZrO_2_):Al_2_O_3_ (1:1)_5_:1 (HZAO)—which means that each odd monolayer of HfO_2_ is replaced by a ZrO_2_ monolayer (1:1) to obtain the same thickness in one supercycle as in the case of a pure HfO_2_ supercycle—were grown by plasma-enhanced atomic layer deposition (PEALD) with ~1 nm for each supercycle on one of a pair of Si wafers using oxygen plasma at a temperature of 250 °C on a FlexAl process tool (OIPT, Yatton, UK) [44].

The wafers with HAO or HZAO layers were implanted with fluences of (2.3–2.5) × 10^16^ cm^−2^ H_2_^+^ ions with an energy of 120 keV for the thermohydride transfer in vacuum after the hydrophilic bonding at temperatures up to 100 °C using a method close to the well-known Smart Cut^®^ technology [45]. After a hydrogen cleavage and transfer of ~500 nm layers of silicon with ferroelectrics to another Si wafer, the annealing was carried out in a furnace in an oxygen atmosphere at 450 °C for 1 h. The finished SFS wafers were subjected to stepwise rapid thermal annealing (RTA) for 30 s in a nitrogen atmosphere up to the temperature of 1000 °C.

**Figure 1:**
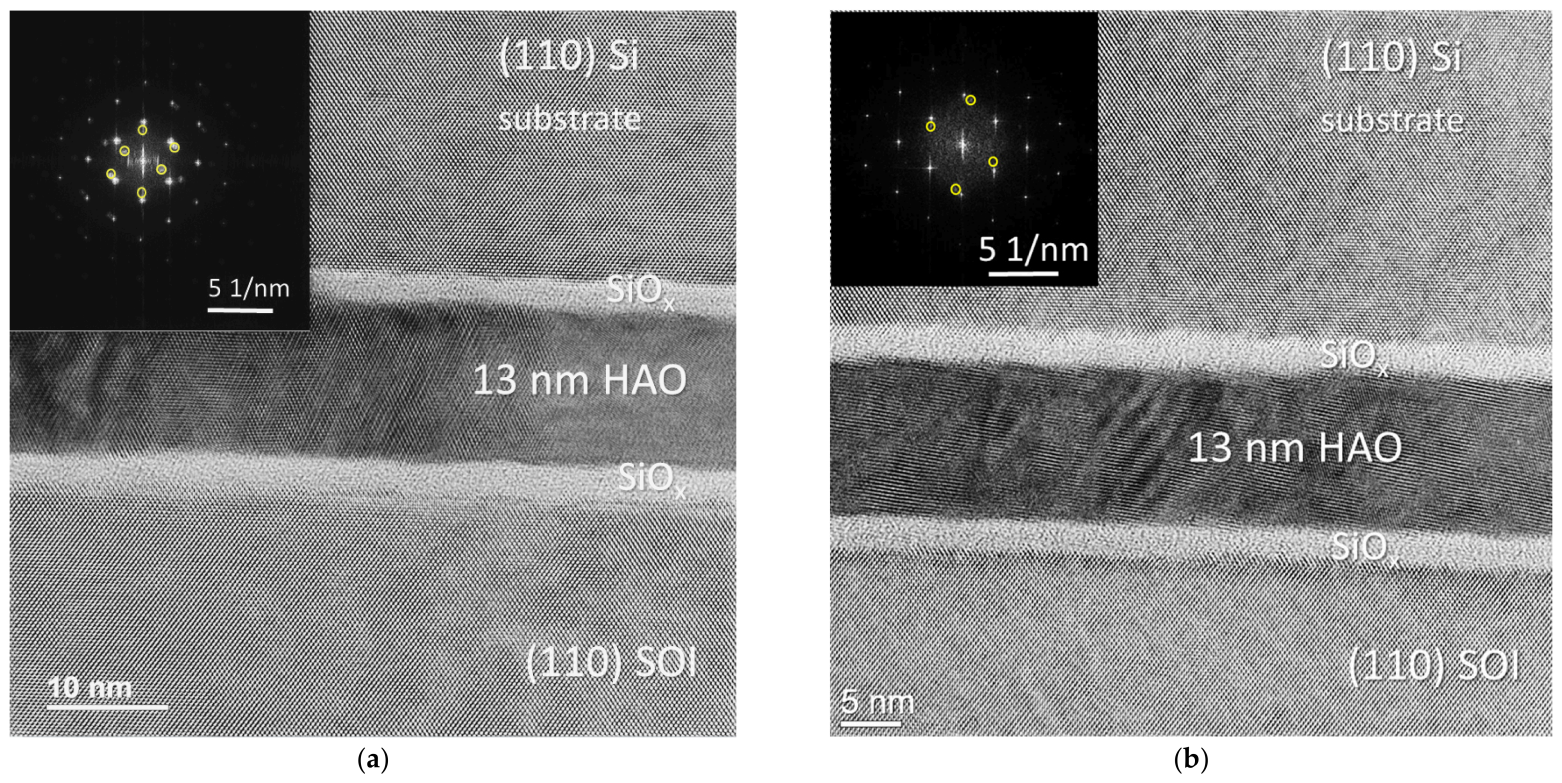
HRTEM images of SFS structures with HAO BOX after the stepwise RTA treatment and upper 1000 °C temperature and two FFT inserts with orthorhombic (**a**) and monoclinic (**b**) phase reflexes marked by yellow circles respectively.

Such RTA treatments ensured the formation of predominantly ferroelectric phases in the buried oxide (BOX) of SFS structures according to the small-angle X-ray diffraction (GIXRD) and high-resolution electron microscopy (HRTEM) data (Figure 1) and unchanged crystal structures during subsequent technological operations [46] and referenced in this one. Thinning the transferred silicon layers to 50 nm was carried out by chemical–mechanical polishing (CMP).

To control the ferroelectric properties of HAO or HZAO nanolaminates, the test structures of MFMS FeFET transistors with a heavily boron-doped polysilicon top layer and 15 nm titanium nitride (TiN) metal (M) electrodes on bulk silicon were also fabricated using the same technology as on SFS substrates. Contact pads sized from 75 to 300 nm to the Si layer were formed by the magnetron sputtering of 100 nm tungsten (W) layers using the lift-off lithography method. Basic matrix crystals on bulk silicon and SFS structures were formed using the standard 1 μm CMOS technology, replacing the furnace heat treatment with the RTA heat treatment at 900 °C.

The measurements of electric characteristics were carried out on wafers with FeFET transistors in probe stations with Keithley 4200A-SCS and Agilent 1500B tools using split-CV, PUND and pseudo-MOSFET methods. The numerical and analytical modeling for the characteristics of devices and integrated circuits was performed at workstations in the academic versions of software TCAD Sentaurus, and LTSPICE.

## 3. Results

### Comparison of Experimental Results with Calculations by TCAD Sentaurus

Figure 1 shows two HRTEM images of SFS structures with a HAO BOX after the stepwise RTA treatment up to 1000 °C temperature with two FFT inserts and orthorhombic and monoclinic phase reflexes marked by circles, respectively. The orthorhombic phase HAO crystallites are rarely observed after such treatment and never for the HZAO layers. The two SiO_x_ interlayers with 2–3 nm thickness are clearly visible.

**Figure 2:**
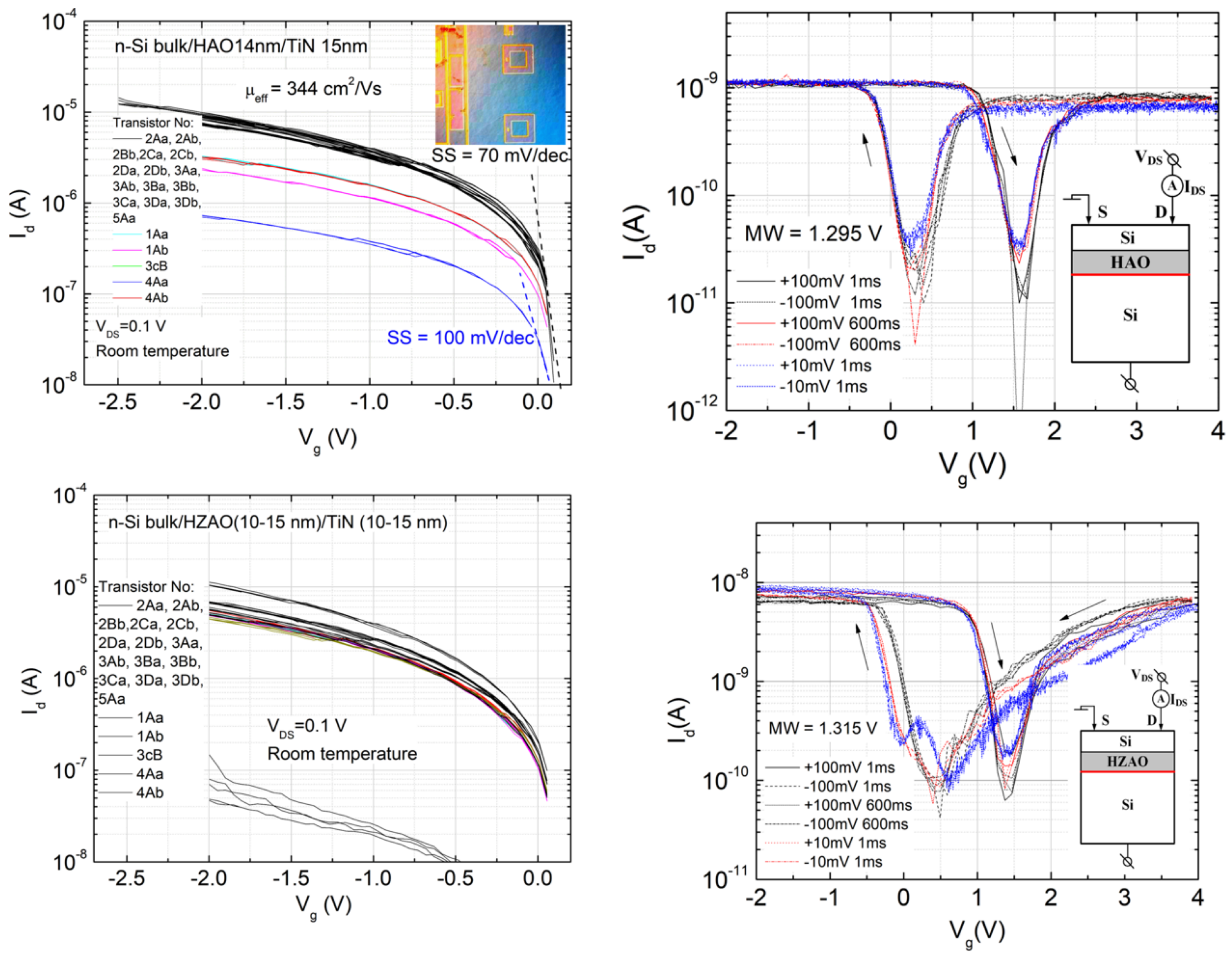
Transfer characteristics (**left**) of twenty ferroelectric *p*-type FeFET transistors fabricated on bulk silicon with 14 nm gate ferroelectrics HAO (**top**) and HZAO (**bottom**). Insert, A photo of a test structure with two transistors; (**right**)—the same for pseudo-FeFET transistors on SFS structures with 20 nm layers of ferroelectrics HAO (**top**) and HZAO (**bottom**) (measurement diagram in the inset) at three different scan rates after the RTA at 900 °C. The arrows present the scan direction. The red line in the insert is an SFS bonding interface.

In Figure 2 are the measured at room temperature transfer characteristics of ferroelectric FeFET test transistors fabricated on bulk silicon and SFS structures in the square geometry with a drain in the center.

On bulk silicon, there is virtually no hysteresis of characteristics due to the inability to repolarize gate ferroelectrics due to the electron leakage current by the injection from the n-type substrate. Pseudo-FeFET transistors with 20 nm layers of HAO and HZAO ferroelectrics exhibit significantly lower leakage currents due to the complete depletion in 50 nm silicon layers after the RTA at 900 °C. As expected, the hysteresis value is slightly higher for the HZAO ferroelectric, but after 950 °C, the hysteresis changes its direction to the opposite one due to a decrease in the proportion of ferroelectric phases and an increase in the density of states at heterointerfaces with silicon, which is in contrast to the HAO SFS structures.

**Figure 3:**
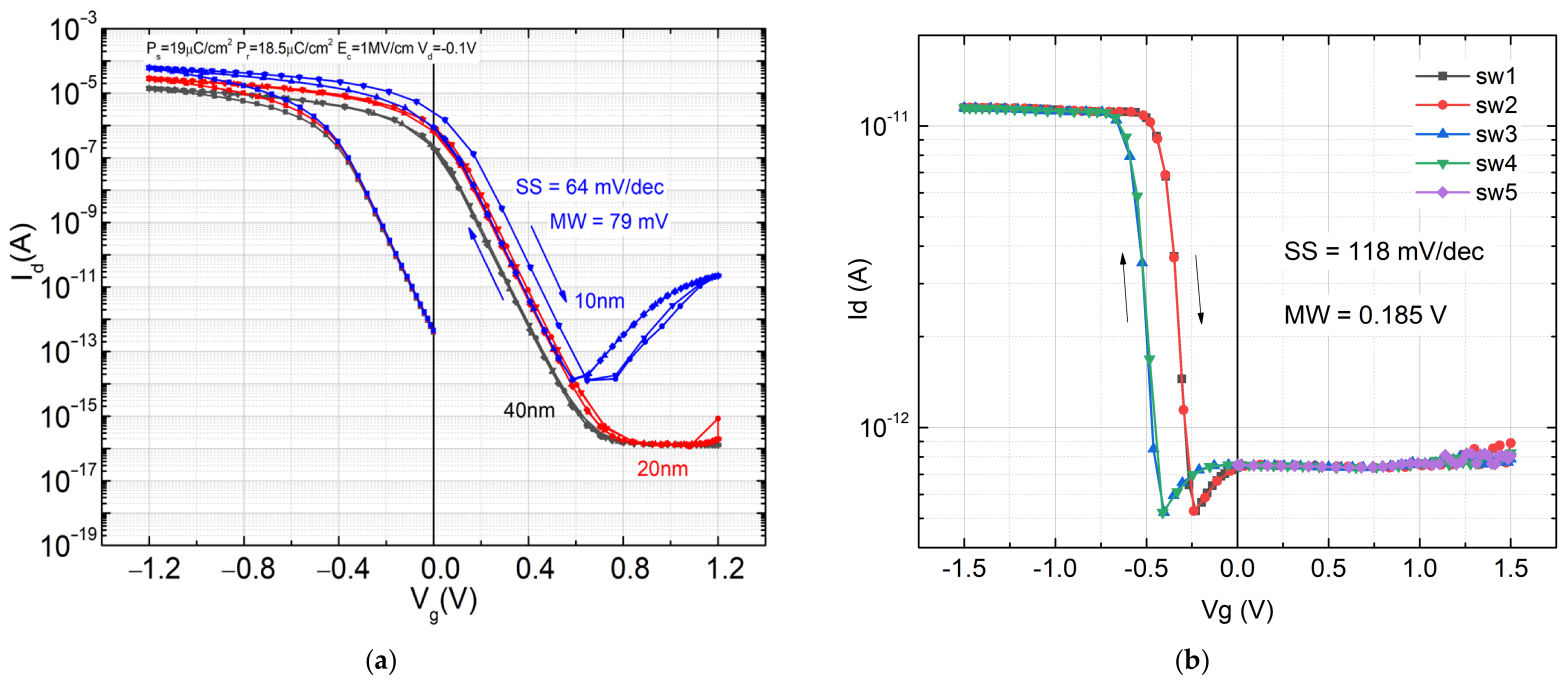
(**a**)—calculated in TCAD transfer characteristics of FeFET with a ferroelectric thickness of 10 to 40 nm and P_r_ = 18.5 µC/cm^2^ on bulk silicon [47]. The curve colors correspond to three marked different thicknesses; (**b**)—the same for the pseudo-FeFET with source-drain tungsten (W) Schottki barriers on the SFS structure with a ferroelectric BOX thickness of 10 nm. The arrows present the scan direction.

The simulation of the FeFET transistor hysteresis in the TCAD Sentaurus software tool was carried out within the Preisach kinetic model of ferroelectrics (Figure 3) [47]. The characteristics of the ferroelectric (E_c_ = 1.2 MV/cm, P_s_ = 18.5 µC/cm^2^ and P_r_ = 18.5 µC/cm^2^) were taken from the experiment [48]. The contributions of charges Q_Fe_ and density of states D_it_ at silicon heterointerfaces with HAO or HZAO ferroelectrics were not taken into account. In contrast to the experiment (Figure 1), a ferroelectric gate of different thickness for bulk silicon and silicon-on-ferroelectric (SFS) structures was located on top. The charge coupling effect for fully depleted transistors (FD FETs) with identical insulator thicknesses shows their equivalence when the gate potential changes [49].

The model for both types of transistors does not exhibit a significant hysteresis when the ferroelectric thickness is 10 nm or more. The small value of the memory window MW (less than 200 mV) for the two types is due to the low supply voltage ±1.5 V with a decreased coercive field to E_c_ = 1.0 MV/cm. To obtain in TCAD simulation the experimental value of MW ~ 1.3 V, we should increase P_r_ to ~390 µC/cm^2^, which seems unreal. An increase in the negative charge density by −1 × 10^11^ cm^−2^ only moves the hysteresis to the right by +0.75 V without MW changing. The leakage is higher and the saturation current of pseudo-FeFETs on the SFS structure is lower, although the MW memory window size is larger as in the experiment, despite some of the voltage drop across the Si substrate and buried high-k oxide (BOX) with two SiO_x_ ILs. The lack of current in the electron-rich mode is a consequence of the smaller size of the space charge region (SCR) at the W contacts of FD FeFETs.

**Figure 4:**
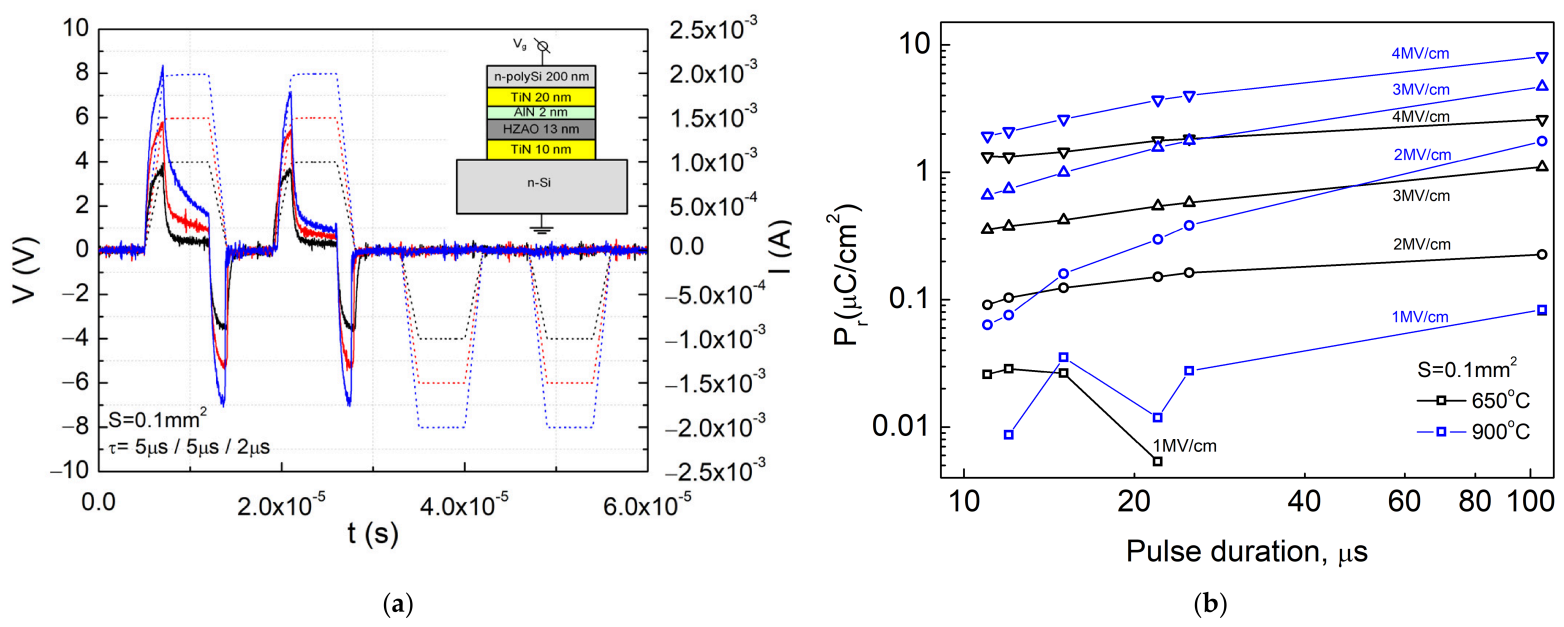
PUND (positive-up and negative-down) pulse measurements of the residual polarization P_r_ dependence on the electrode voltage V for an MFMS FeCAP HZAO structure (**a**) and the dependence of P_r_ on the electric field E(V) and pulse duration in µs for two RTA temperatures (**b**). The curve colors correspond to three marked by dashed lines different pulse voltages.

The ferroelectric properties of HAO or HZAO nanolaminated layers were measured on MIFMS FeFET structures (Figure 4). They are an integral analogue of 1T-1C FRAM memory cells with ferroelectric capacitance FeCAP included in the drain circuit or in the gate circuit [6,7,46]. The upper and lower layers of the TiN metal served as electrical contacts and, at the same time, barriers for the oxygen diffusion from ferroelectrics HAO or HZAO during stepwise RTA treatments. Using C-V and PUND (positive-up and negative-down) pulse measurements, the dependences of the residual polarization on the electrode voltage Pr (V) and the time characteristics of recharge pulses dV/dt, as well as on their duration, were determined (Figure 4b).

It is shown in Figure 4 that even with a 15 nm thickness of metal TiN electrodes, it is not possible to ensure the symmetrical repolarization at the TiN contacts to the ferroelectric HZAO layer due to the absence of bias currents and complete repolarization on the PUND pulse sequence, but it is possible only to reach P_r_ = 9 µC/cm^2^ at the maximum field E = 4 MV/cm. The obtained value of P_r_ is two times less than the value accepted in the calculations, which is P_r_ = 18.5 µC/cm^2^ according to [48]. A low P_r_ value can be due to the additional 2 nm AlN interlayer placed by PEALD to avoid TiN oxidation in the CMOS industrial process. An increase in the E(V) field to 4 MV/cm leads to an increase in P_r_ by more than one order of magnitude to ensure a quantitative agreement between the calculated models and the measurement results for HAO and HZAO pseudo-FeFET transistors on SFS structures (Figure 4b). It has not yet been possible to increase the calculated field E to 4 MV/cm in the TCAD modeling due to the need to increase the nodes of the computational grid and a sharp increase in the simulation time on the workstation. It was also not possible to obtain a quantitative correspondence by simply changing the upper and lower gate oxide thicknesses of DG FeFET transistors.

## 4. Compact Models of FeFET Transistors for an Analog Content-Addressable Memory Based on Crossbar

The compact FeFET crossbar models used in circuit analysis systems such as Verilog-A enable the prediction of FeFET crossbar characteristics and validation of their suitability for solving AI problems. Such models were used in the BSIM-IMG software for describing DG SOI FD FET transistors [46,48,49,50,51]. These models lacked a multi-level description of analog polarization switching.

Based on the gate-drain current–voltage characteristic, a SPICE model of the DG FeFET was developed as an analytical approximation of the current–voltage characteristics. This SPICE model was used to create a crossbar model that can be used as a content addressable memory (CAM). In the CAM, search inputs are compared against a stored data table, and the address of the found data is returned. This approach utilizes in-memory analog computations on the crossbars of non-volatile DG FeFET memory cells. This results in low power consumption and eliminates the need for digital-to-analog converters. In this new microcircuit architecture, information is stored and processed in the same locations (in memory circuits) [52] as in the synapses of neurons in the brain.

The production of arrays of dual-gate ferroelectric transistors (DG FeFETs) on a silicon substrate (SFS) will enable the design of signal transmission systems and will enable the transition from simple crossbars to deep and recurrent neural networks. Information storage and processing with ultra-low power consumption occurs simultaneously and in parallel in all cells. Mathematical electronic device models exist that are based on a detailed physical description of the device. In the absence of such a description, phenomenological models based on the approximation of the device’s behavior curve—for example, its current–voltage characteristic—are used. Examples of such models include memristor models [53,54,55,56,57,58].

In von Neumann processors, the bus between the main memory and the logic units is a bottleneck, increasing data transfer latency and power consumption. To address this problem, a memory-based computing paradigm [52] was proposed that is based on one of the fundamental brain features—computing in memory elements (synapses). Memory-based computing systems consist of memory cells used to perform logical and arithmetic operations. The fabrication of SFS arrays from double-gate ferroelectric transistors (DG FeFETs) will enable the development of weight storage matrices for neuromorphic computing [46]. Content-addressable memory (CAM) compares search input data with a stored data table and returns the searched data address. Furthermore, the memory-based computing architecture using DG FeFETs provides ultra-low power consumption.

### 4.1. DG FeFET SPICE Model Based on the Gate-Drain Current-Voltage Characteristic

**Figure 5:**
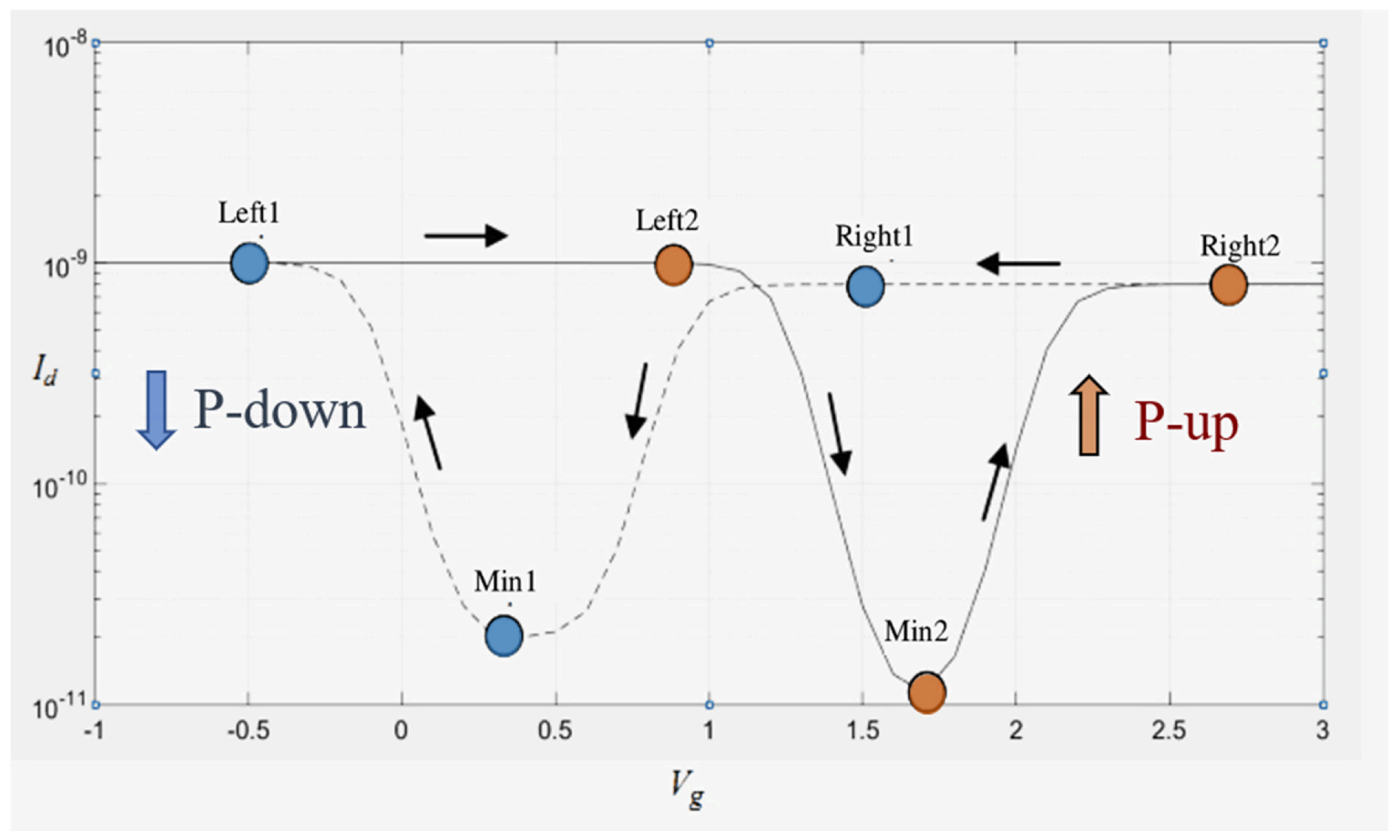
Model of the current–voltage characteristic of a double-gate ferroelectric transistor with two ferroelectric polarizations adapted from Figure 2. The arrows indicate the scan direction.

Examples of phenomenological models include numerous models [54,55]. Figure 5 shows a SPICE model of the gate-drain current–voltage characteristic of a ferroelectric transistor (DG FeFET). Six singular points at the current–voltage characteristic can be distinguished in Figure 5. Point Min1 is the point of the local minimum of the characteristic (dashed line) obtained when the gate voltage $V_{g}$ goes down. At point Left1, this characteristic comes to the asymptotic horizontal to the left of the minimum point, Min1. At point Right1, the characteristic comes to the asymptotic horizontal to the right of the minimum point Min1. Similarly, 3 special points—Left2, Min2, Right2—are selected for the gate voltage rising (solid line). The indicated 6 points of the DG FeFET current–voltage characteristic can be used to construct its analytical description. The arrows show the voltage change direction (increase or decrease). This direction determines the choice of one of the two curves for changing the drain current.

From Figure 5, it follows that the two drain current $I_{d}$ curves (dashed and solid) approximately coincide at gate voltages $V_{g}<V_{Left1}$ and $V_{g}>V_{Right2}$. At these voltages, the current–voltage characteristic switches from one curve to the other if the direction of the gate voltage changes from increasing to decreasing. The corresponding model is implemented in the LTSPICE simulation system [56]. The asymptotic values of the drain currents are equal to *I_Left_*_1_ and *I_Right_*_2_, respectively. As the gate voltage $V_{g}$ decreases, the drain current line passes through the local minimum point Min1, and as the gate voltage $V_{g}$ increases, it passes through the point Min2. Four fragments of the sigmoid function

(1) $$\sigma (x)=\frac{1}{1+e^{-\beta x}}$$

are used to approximate the current–voltage characteristic. For $x\in [V_{Min1},V_{Right1}]$, we will seek a characteristic in the form of a scaled sigmoid.

(2) $$I(x)=\;a_{Right1}\cdot \sigma (x-c_{Right1})+b_{Right1}$$

Here, the sigmoid increases from $I(V_{Min1})$ to $I(V_{Right1})$. We take point

(3) $$c_{Right1}=(V_{Min1}+V_{Right1})/2$$

as the center of the sigmoid. Assuming $\sigma (V_{Min1}-c_{Right1})=0$ from (2), we obtain

(4) $$b_{Right1}=I(V_{Min1})=I_{Min1}$$

Substituting $x=V_{Right1}$ into (2), taking into account (3) and (4), we obtain

$$I_{Right1}=I(V_{Right1})=\;a_{Right1}\cdot \sigma (V_{Right1}-c_{right1})+I_{Min_{1}}.$$

Hence,

(5) $$a_{Right1}=\frac{I_{Right1}-I_{Min1}}{\sigma (V_{Right1}-c_{Right1})}$$

For $x\in [V_{Left1},V_{Min1}]$, assuming $I(x)=\;a_{Left1}\cdot \sigma (-(x-c_{Left1}))+b_{Left1}$, we obtain

$$c_{Left1}=(V_{Min1}+V_{Left1})/2,\;\;b_{Left1}=I_{Min1},$$

(6) $$a_{Left1}=\frac{I_{Left1}-I_{Min1}}{\sigma (V_{Min1}-c_{Left1})}.$$

Accordingly, for $x\in [V_{Min2},V_{Right2}]$ and $x\in [V_{Left2},V_{Min2}]$,

(7) $$a_{Right2}=\frac{I_{Right2}-I_{Min2}}{\sigma (V_{Right2}-c_{Right2})},$$

$$c_{Left2}=(V_{Min2}+V_{Left2})/2,\;\;b_{Left2}=I_{Min2},$$

(8) $$a_{Left2}=\frac{I_{Left2}-I_{Min2}}{\sigma (-(V_{Min2}-c_{Left2})}.$$

As a result, the following formulas describing the hysteresis curve were obtained:

(9) $$I_{d}(V_{g})=\left\{\begin{array}{c} I_{Min1}+(I_{Left1}-I_{Min1})\frac{\sigma (-(V_{g}-c_{Left1}))}{\sigma (-(V_{Left1}-c_{Left1}))},\;\;V_{g}<V_{Min1},\\ I_{Min1}+(I_{Right1}-I_{m_{1}})\frac{\sigma (V_{g}-c_{Right1})}{\sigma (V_{Right1}-c_{Right1})},\;\;V_{g}\geq V_{Min1}. \end{array}\right.$$

(10) $$I_{d}(V_{g})=\left\{\begin{array}{c} I_{Min2}+(I_{Left2}-I_{Min2})\frac{\sigma (-(V_{g}-c_{Left2}))}{\sigma (-(V_{Left2}-c_{Left2}))},\;\;V_{g}<V_{Min2},\\ I_{Min2}+(I_{Right2}-I_{Min2})\frac{\sigma (-(V_{g}-c_{Right2}))}{\sigma (-(V_{Right2}-c_{Right2}))},V_{g}\geq V_{Min2}, \end{array}\right.$$

where

$$c_{Left1}=\frac{V_{Left1}+V_{Min1}}{2},\;\;c_{Right1}=\frac{V_{Right1}+V_{Min1}}{2},\;\;c_{Left2}=\frac{V_{Left2}+V_{Min2}}{2},\;\;c_{Right2}=\frac{V_{Right2}+V_{Min2}}{2}.$$

The hysteresis curve in Figure 5 was calculated in MATLAB using Formulas (9) and (10) with the above-mentioned values of the parameters of the special points of the current–voltage characteristic and the value of β=16. The analytical model well approximates the current–voltage characteristic at the special points Left1,Min1,Right1,Left2,Min2,Right2.

The corresponding SPICE model is implemented in LTspice [59] and describes a device with terminals G, S, D (the gate, source, and drain) of the FeFET transistor. The switching between the curves (9) and (10) specified above is realized by a Schmitt trigger with parameters *Vt* и *Vh*. Initially, the voltage at the Schmitt trigger output is zero. When the trigger input voltage exceeds the value Vt+Vh, the voltage at the trigger output becomes Vhigh>0 (“logic one”), and the GSD circuit switches from curve (9) to curve (10). The Schmitt trigger remains in the high state until the input voltage drops to the value *Vt* − *Vh*. In this case, the voltage *Vhigh* at the trigger output becomes zero (“logic low”), and the GSD circuit switches from curve (10) to curve (9). To ensure that the curves switch at given values of $V_{Left1}$ and $V_{Right2}$, the Schmitt trigger parameters *Vt* and *Vh* must be selected such that the equalities

$$Vt-Vh=V_{Left1},\;\;Vt+Vh=V_{Right2},$$

hold, i.e., $Vt=(V_{Left1}+V_{Right2})/2$, $Vh=(V_{Right2}-V_{Left1})/2$, where *Vhigh* = 1.0 V, *Vt* = 1.0 V, *Vh =* 1.5 V.

The drain current is initially described in Figure 5 by a solid line (mode 1). Let the gate voltage $V_{g}$ be $V_{Left1}$ (first stage). Then, the drain current is equal to $I_{Left1}$. If we increase the gate voltage to $V_{Min2}$ (second stage), then the drain current drops to $I_{Min2}$. When the voltage $V_{g}\geq V_{Right2}$ is applied to the gate (stage 3), the transistor switches to mode 2 (dashed line). In this case, the drain current increases to $I_{Right2}$. If, in mode 2, we decrease the gate voltage to $V_{Min1}$ (stage 4), then the drain current drops to $I_{Min1}$. Switching back to mode 1 occurs by applying a voltage $V_{g}\leq V_{Left1}$ to the gate. In this case, the drain current increases to $I_{Left1}$. So, the drain current versus gate voltage dependence is consistent with the transfer characteristic of DG FeFET (Figure 5).

### 4.2. SPICE Model of a DG FeFET Crossbar

**Figure 6:**
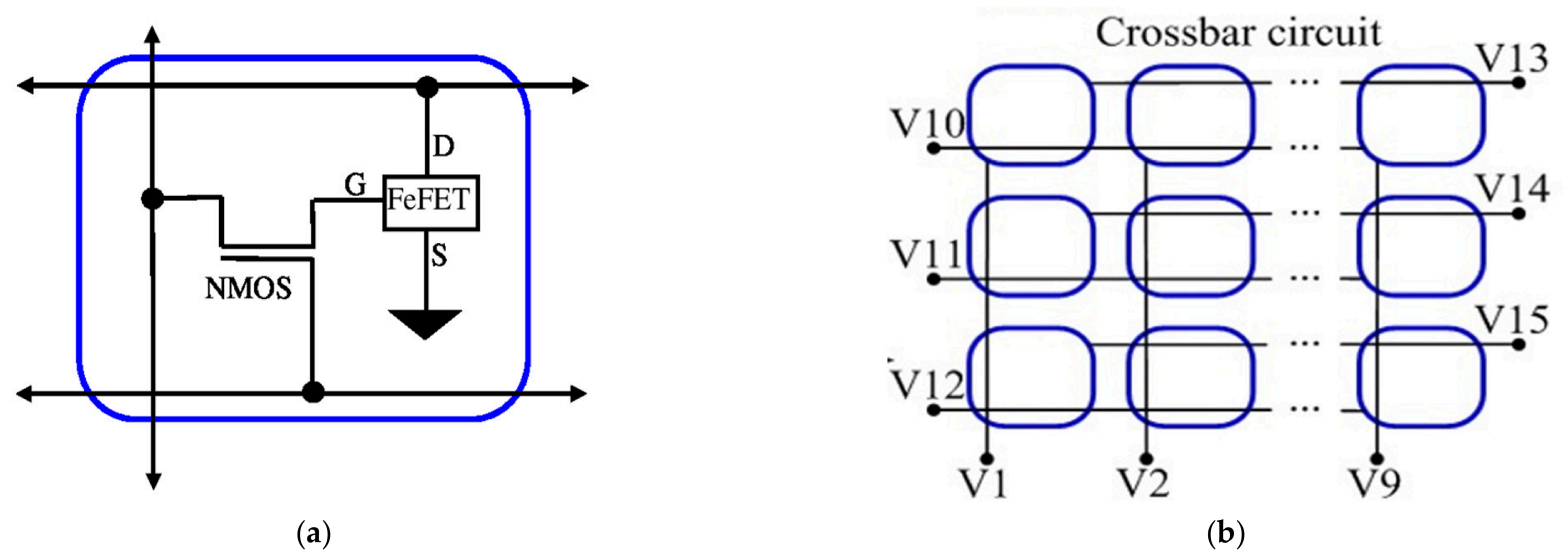
(**a**)—Example of a crossbar cell, where NMOS is the logic n-type FET, G is the gate, S is the source, and D is the drain of a FeFET; (**b**)—example of a crossbar circuit with Figure 6a cells.

A software model of content-addressable (associative) memory (CAM) has been developed in the LTspice environment based on a FeFET crossbar model with analog input processing. The developed model enables the following:

1. Loading reference vectors into the CAM;
2. Comparing input vectors with analog component values and reference vectors stored in the CAM. A crossbar consisting of cells (Figure 6a) is proposed as the CAM (Figure 6b).

The cell includes a DG FeFET transistor (G is the front gate, S is the source, D is the drain, and the back gate is grounded) and a logic NMOS field effect transistor as well as a switch that turns off the FeFET gate. This design allows the state of any FeFET to be independently changed, thus storing reference vectors in the crossbar rows (content-addressable memory). In Figure 6, the vertical bus receives the input signal of a crossbar cell, and the horizontal bus receives the signal that controls the switch.

Figure 6b shows an example of a crossbar circuit that stores three reference weight vectors of dimension N=9. Each crossbar row calculates the activation of one neuron (the total drain current of the transistors in the row) as the product of the input data vector and the neuron weight vector, which is represented by the conductance of the corresponding FeFET transistors.

The binary images of the symbols L,T,X and the distorted symbols L*, T*, X* can be described by matrices:

$$L=\left(\begin{array}{ccc} 1 & 0 & 0\\ 1 & 0 & 0\\ 1 & 1 & 1 \end{array}\right),\;\;\;\;T=\left(\begin{array}{ccc} 1 & 1 & 1\\ 0 & 1 & 0\\ 0 & 1 & 0 \end{array}\right),\;\;X=\left(\begin{array}{ccc} 1 & 0 & 1\\ 0 & 1 & 0\\ 1 & 0 & 1 \end{array}\right),$$

$$L^{*}=\left(\begin{array}{ccc} 1 & 0 & 0\\ 1 & 0 & 1\\ 0 & 1 & 1 \end{array}\right),\;\;\;\;T^{*}=\left(\begin{array}{ccc} 1 & 1 & 1\\ 0 & 1 & 0\\ 0 & 0 & 0 \end{array}\right),\;\;X^{*}=\left(\begin{array}{ccc} 1 & 0 & 1\\ 0 & 1 & 1\\ 0 & 0 & 1 \end{array}\right)$$

Here, 0 is a black pixel and 1 is a white pixel. Then, the images can be described as vectors (row-by-row scanning of the images).

These vectors can be written to the crossbar as DG FeFET conductance vectors by supplying voltage vectors (in volts) to the crossbar input (voltage sources *V*1, *V*2, …, *V*9 in Figure 6b) correspondingly:

$$V_{L,write}=\;(3,\;-1,\;-1,\;3,\;-1,\;-1,\;3,\;3,\;3),\;V_{T,write}=\;(3,\;3,\;3,\;-1,\;3,\;-1,\;-1,\;3,\;-1)$$

$$V_{X,write}=\;(3,\;-1,\;3,\;-1,\;3,\;-1,\;3,\;-1,\;3)$$

The switches that allow data input into the crossbar rows are controlled by voltage sources *V*10, *V*11, and *V*12. When recording reference vectors (programming the crossbar), these sources work sequentially. When processing data (testing the programmed crossbar), all these sources allow data input into the crossbar rows at the same time.

**Figure 7:**
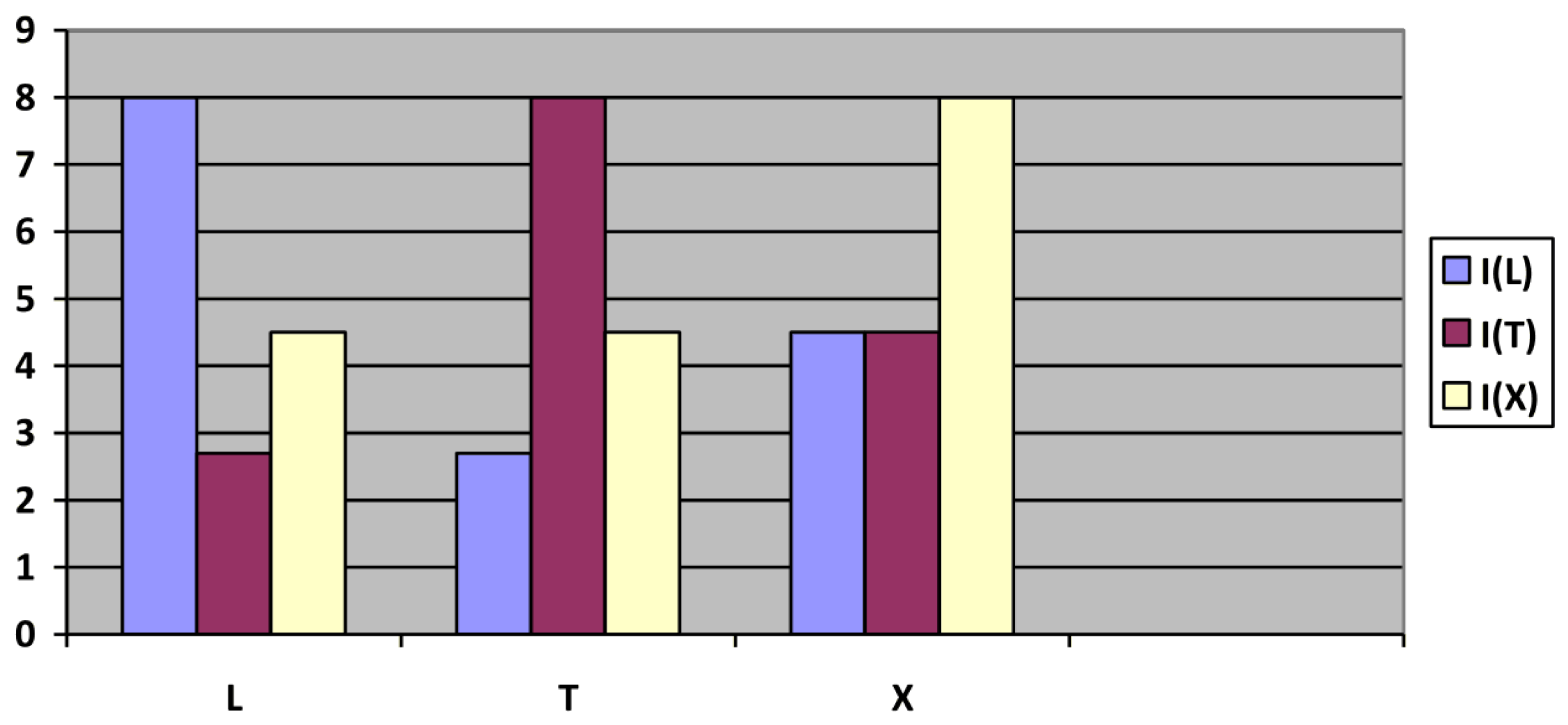
The dependence of the crossbar output currents *I*(*L*), *I*(*T*) and *I*(*X*) (nA) on the input images L, T, and X.

The DG FeFET is set to mode 2 (dashed line in Figure 5) by the voltage of 3 volts (input of white pixel) and to mode 1 (solid line) by the voltage of −1 volts (input of black pixel). Next, the voltage vectors

$$V_{L}=(V_{m_{2}},V_{m_{1}},V_{m_{1}},V_{m_{2}},V_{m_{1}},V_{m_{1}},V_{m_{2}},V_{m_{2}},V_{m_{2}}),$$

$$V_{T}=(V_{m_{2}},V_{m_{2}},V_{m_{2}},V_{m_{1}},V_{m_{2}},V_{m_{1}},V_{m_{1}},V_{m_{2}},V_{m_{1}}),$$

$$V_{X}=(V_{m_{2}},V_{m_{1}},V_{m_{2}},V_{m_{1}},V_{m_{2}},V_{m_{1}},V_{m_{2}},V_{m_{1}},V_{m_{2}})$$

modeling images of symbols *L*, *T*, *X*, are fed sequentially to the input of the crossbar, where a white image pixel corresponds to voltage $V_{m_{2}}=V_{Min2}\approx 1.7\;\;V$, and voltage $V_{m_{1}}=V_{Min1}\approx 0.3\;\;\;V\;$ corresponds to a black image pixel. These voltages provide the maximum output current when the input vector matches the reference and the minimum output current if it does not: $V_{m_{1}}$ for mode 1 and $V_{m_{2}}$ for mode 2 (Figure 7).

Consider the distorted input vectors:

$$V_{L}^{*}=(V_{m_{2}},V_{m_{1}},V_{m_{1}},V_{m_{2}},V_{m_{1}},V_{m_{2}},V_{m_{1}},V_{m_{2}},V_{m_{2}}),$$

$$V_{T}^{*}=(V_{m_{2}},V_{m_{2}},V_{m_{2}},V_{m_{1}},V_{m_{2}},V_{m_{1}},V_{m_{1}},V_{m_{1}},V_{m_{1}}),$$

$$V_{X}^{*}=(V_{m_{2}},V_{m_{1}},V_{m_{2}},V_{m_{1}},V_{m_{2}},V_{m_{2}},V_{m_{1}},V_{m_{1}},V_{m_{2}}).$$

**Figure 8:**
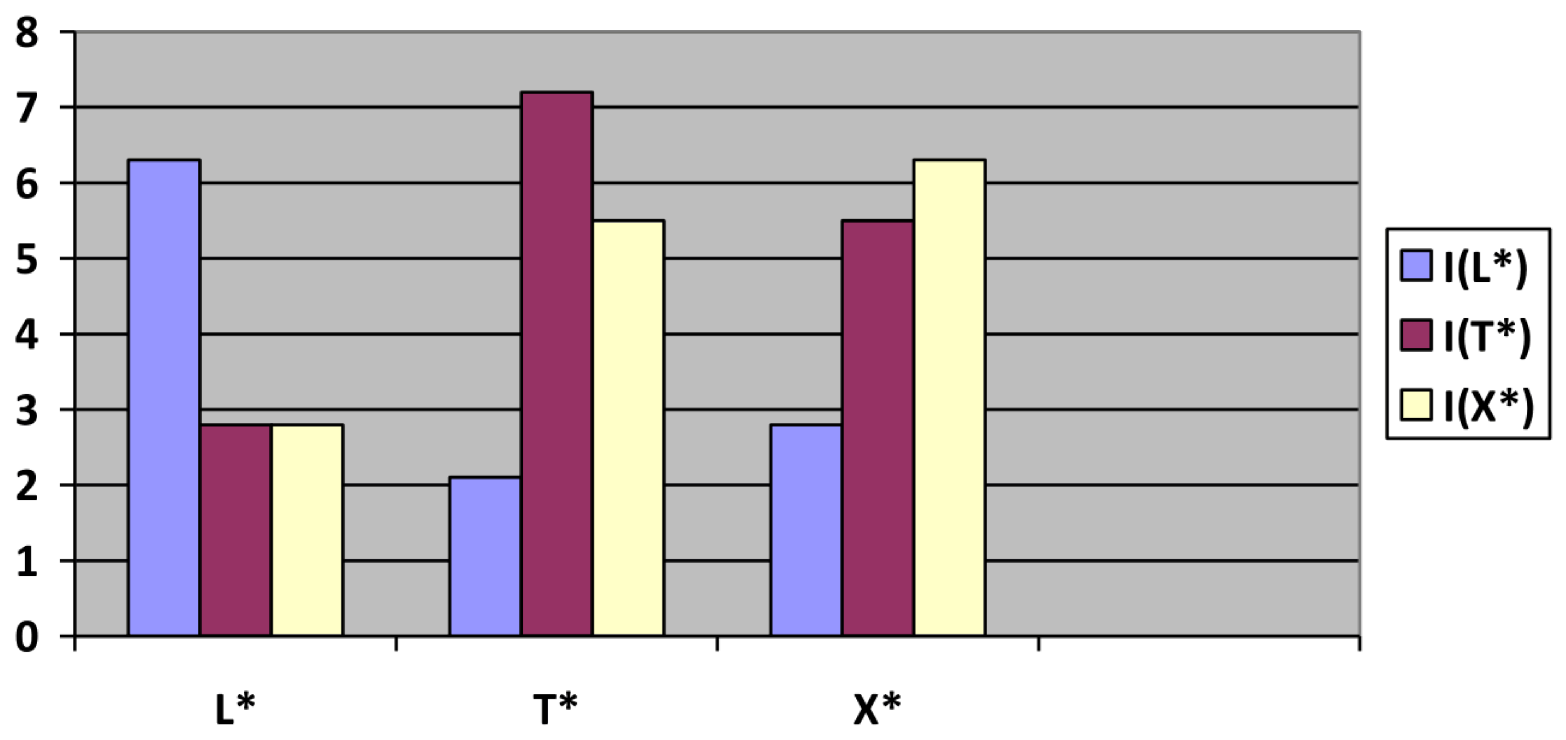
The dependence of the crossbar output currents *I*(L*), *I*(T*) and *I*(X*) (nA) on the input images L*, T*, and X*.

Figure 8 shows that when distorted images are supplied, the output currents $I_{L},I_{T},I_{X}$ ratios change quantitatively, but they do not change qualitatively—that is, the maximum currents correspond to the reference vectors stored in the crossbar, which confirms the possibility of using it as CAM (associative memory).

The described crossbar is an analog device and does not require a digital-to-analog converter to input information. In this regard, it is of interest to answer the question of how much the input voltage $V_{g}$ can deviate from the voltages $V_{m1}$ and $V_{m2}$, respectively.

Let us represent the deviation in the form $d=\delta \cdot (V_{m2}-V_{m1})$, where δ is the parameter specifying the deviation. In vectors $V_{L},V_{T},V_{X}$, we replace $V_{m_{2}}$ by $V_{m_{2}}-d$ and $V_{m_{1}}$ by $V_{m_{1}}+d$, and as a result, we obtain vectors $V_{L}^{’},V_{T}^{’},V_{X}^{’}$. Modeling with input vectors $V_{L}^{’},V_{T}^{’},V_{X}^{’}$ has shown that for the increase in δ from zero to 0.4999, the difference between the maximum currents and the rest decreases, and at δ=0.4999, it is at least $10^{-3}$ nA. Similar relationships between currents are preserved when δ decreases from zero to − 0.576. Thus, the permissible deviations of the input voltages are about 50% of the voltage difference $V_{m1}$ and $V_{m2}$.

## 5. Conclusions

The novelty and significance of this paper are the establishment of the thermal stability of ferroelectric HAO and HZAO buried oxides in an industrial CMOS process with RTA thermal treatments up to 900 °C as gate insulators for fully depleted dual-gate field effect transistors on SFS structures.

Namely, the HAO and HZAO SFS structures are proposed as CMOS compatible and thermally stable substrates for energy-efficient low-power integral circuits with inherent NVM cells based on DG FD FeFETs. The TCAD Sentaurus modeling presents only the qualitative coincidence with the experimentally measured characteristics. The account of ferroelectric charge and interface state density are needed to improve the physical models.

A SPICE model of the crossbar-based content-addressable memory is proposed using an analytical model of the gate-drain current–voltage characteristic for DG FeFET. It is shown that the following apply:

(1) The maximum is the output current of the crossbar row that stores the reference corresponding to the input vector and decreases as the distance between the input vector and the reference vector increases;
(2) The crossbar is able to recognize distorted vectors corresponding to the reference vector (associative memory property);
(3) The limits of the input voltage change are determined, at which the crossbar distinguishes distorted reference images.

## Data Availability

The original contributions presented in this study are included in the article. Further inquiries can be directed to the corresponding author.

## Abbreviations

The following abbreviations are used in this manuscript:

SOI	Silicon-on-insulator
SFS	Silicon–ferroelectric–silicon
BOX	Buried oxide
IL	Interlayer
FeFET	Ferroelectric field effect transistor
SCR	Space charge region
FD	Fully depleted
MFM	Metal–ferroelectric–metal
MFS	Metal–ferroelectric–semiconductor
MFIS	Metal–ferroelectric–insulator–semiconductor
DG	Double-gate
NVM	Non-volatile memory
ACAM	Analog content addressable memory
